# Bilateral Diaphragmatic Paralysis in a Patient With Critical Illness Polyneuropathy: A Case Report: Erratum

**DOI:** 10.1097/01.md.0000471622.52003.5a

**Published:** 2015-09-11

In the article “Bilateral Diaphragmatic Paralysis in a Patient With Critical Illness Polyneuropathy: A Case Report”,^1^ which appeared in Volume 94, Issue 31 of *Medicine*, several values in Tables 1 and 2 were either not included or appeared in incorrect columns. Also, the affiliations footnote contained multiple errors. The article has since been corrected online.
